# P21-activated kinase 4 involves TSH induced papillary thyroid cancer cell proliferation

**DOI:** 10.18632/oncotarget.15079

**Published:** 2017-02-04

**Authors:** Xiaochen Xie, Xiaoguang Shi, Haixia Guan, Qiqiang Guo, Chenling Fan, Wenwu Dong, Guiling Wang, Feng Li, Zhongyan Shan, Liu Cao, Weiping Teng

**Affiliations:** ^1^ Department of Endocrinology and Metabolism, Institute of Endocrinology, Liaoning Provincial Key Laboratory of Endocrine Diseases, The First Affiliated Hospital of China Medical University, China Medical University, Shenyang, P.R. China; ^2^ Key Laboratory of Medical Cell Biology, College of Translational Medicine, China Medical University, Shenyang, P.R. China; ^3^ Department of Thyroid Surgery, The First Affiliated Hospital of China Medical University, Shenyang, P.R. China; ^4^ Department of Cell Biology, Key Laboratory of Cell Biology, Ministry of Public Health, and Key Laboratory of Medical Cell Biology, Ministry of Education, China Medical University, Shenyang, P.R. China

**Keywords:** p21-activated kinase 4, thyroid-stimulating hormone, PKA Cα, papillary thyroid cancer, cell proliferation

## Abstract

Papillary thyroid cancer is a common endocrine malignancy. Although p21-activated kinase 4 (PAK4) is involved in the development of different types of tumor, its function has not been investigated in papillary thyroid cancer. Here, we identified a role for PAK4 in papillary thyroid cancer progression. Levels of PAK4 and PAK4 phosphorylated at serine 474 correlated significantly with tumor size and TNM stage. Furthermore, stable knockdown of PAK4 retarded cellular proliferation, migration, and invasion. Moreover, thyroid stimulating hormone-induced cellular proliferation in papillary thyroid cancer was found to be dependent on TSHR/cAMP/PKA/PAK4 signaling, with levels of phosphorylated PAK4 correlating positively with serum thyroid stimulating hormone and PKA Cα levels in patients with papillary thyroid cancer. These findings revealed a novel function of PAK4 in thyroid stimulating hormone-induced papillary thyroid cancer progression and suggest that PAK4 may become a promising diagnostic and therapeutic target for this disease.

## INTRODUCTION

Thyroid cancer is one of the most common endocrine malignancies, with incidence rates predicted to increase by the year 2030 to make it the fourth leading cancer in terms of diagnosis [1]. Papillary thyroid carcinoma (PTC) is usually indolent and curable, but it can spread early to local lymph nodes. In addition, disease persistence and recurrence have been associated with increased mortality in PTC [2]. Therefore, proteins that regulate PTC development and progression may act as novel prognostic markers and have to be identified to improve prognosis.

The p21-activated kinases (PAKs) belong to a conserved family of serine/threonine protein kinases. Till date, the six mammalian PAKs have been classified into two groups: group I (PAK1–3) and group II (PAK4–6) [3]. PAK4 activity can be induced in a Rho GTPase-dependent or -independent manner. Additionally, PAK4 phosphorylated at serine 474 (p-PAK4) is considered as the form of kinase activation [4]. PAK4 performs several functions: protection from apoptosis [5], promotion of microtubule dynamics [6–8], inhibition of cell adhesion [9–11], maintenance of stem cell-like phenotypes [12], induction of cell proliferation [13], promotion of cell migration [14–17], regulation of tumorigenesis [18, 19], and promotion of anchorage-independent growth [20]. Furthermore, PAK4 overexpression has been detected in thyroid cancer, cell lines derived from breast, prostate, gall bladder, stomach and ovarian cancers, as well as in several primary tumors [20–25]. Mounting evidence suggest that PAK4 expression is tightly correlated with cancer progression, which makes PAK4 a potentially promising diagnostic and therapeutic target for cancer therapy.

Thyroid-stimulating hormone (TSH) plays a key role in the development of clinically detectable thyroid cancer [26]. After binding to the thyroid stimulating hormone receptor (TSHR), TSH activates cAMP production [27, 28], which is responsible for the maintenance of thyroid hormone secretion and thyroid cell proliferation [29]. In differentiated thyroid cancers, TSH stimulation is associated with cancer cell growth, whereas TSH suppression is associated with growth inhibition, suggesting that differentiated thyroid carcinomas are TSH-dependent tumors [30, 31].

In this study, we investigated the role of PAK4 in PTC progression. Our findings suggest that TSH-induced cellular proliferation in PTC is dependent on TSHR/cAMP/PKA/PAK4 signaling.

## RESULTS

### Upregulation of PAK4 and p-PAK4 in PTC and correlation of their levels with clinical features

**Figure 1:**
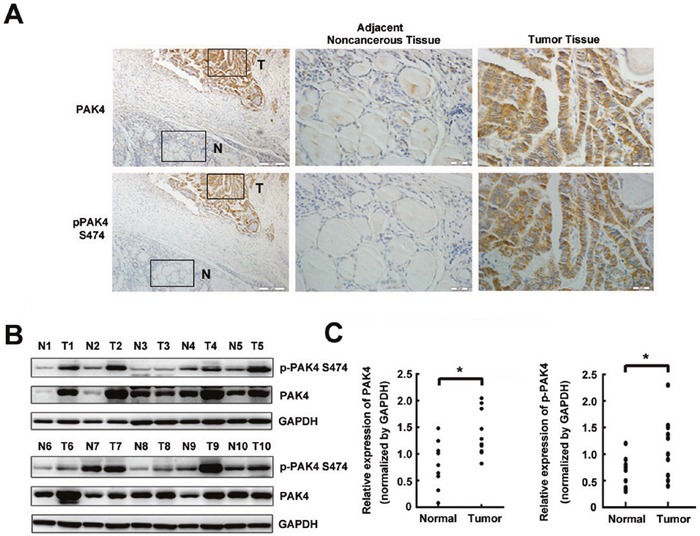
PAK4 and p-PAK4 levels in PTC samples **A**. Representative immunohistochemical staining images. The boxed areas in the left hand side images are magnified in the middle and right hand side panels, with adjacent noncancerous tissue (N; shown in the middle column) and tumor tissue (T; shown in the right hand side column) displayed at a magnification of 100×. **B**. p-PAK4 at serine 474 and PAK4 levels were analyzed by western blot analysis. **C**. p-PAK4 at serine 474 and PAK4 data (B) visualized via scatter diagram. * *P* < 0.05.

To identify the roles of PAK4 and p-PAK4 in the development and progression of PTC, their levels were assessed by immunohistochemistry in normal thyroid and PTC tissue specimens (n = 98) (Figure 1A). The results showed that 90.8% of tumor samples and 9.2% of normal thyroid tissue samples showed positive PAK4 staining, while 82.7% of tumor samples and 15.3% of normal thyroid tissue samples were positive for p-PAK4 (Table 1). Additionally, when PAK4 and p-PAK4 levels were compared between paired samples from same patients by western blotting, elevated levels of p-PAK4 and PAK4 were observed in PTC samples than in their normal paired counterparts (Figures 1B and 1C). Further analysis revealed that the upregulation of PAK4 and p-PAK4 in PTC samples was significantly associated with tumor size (all *P* < 0.01) and tumor TNM stage (all *P* < 0.01; Table 2). No correlations were found with gender, patient age, or lymph node metastasis. These findings suggest that levels and activity of PAK4 correlate with the stage of PTC.

**Table 1:** Expression of p-PAK4 ser474, PAK4 and PKA Cα in PTC and adjacent normal tissues

	p-PAK4 ser474				PAK4				PKA Cα			
	-	+	++	+++	-	+	++	+++	-	+	++	+++
PTC (n=98)	17	32	28	21	9	38	32	19	12	37	34	15
Adjacent normal tissues (n=98)	83	11	4	0	89	7	2	0	80	13	6	0

**Table 2:** Correlation of expression levels of PAK4 and PAK4 phosphorylation at Ser474 with clinicopathological features in PTC

	Case(n)	PAK4 fold^a^	P-value	p-PAK4 Ser474 fold^a^	P-value
Gender					
Male	27	4.6(2.5-7.2)	0.414	5.2(2.5-7.3)	0.650
Female	71	5.8(2.5-7.8)		5.0(2.4-8.8)	
Age					
≤ 45	55	4.9(2.5-7.2)	0.772	4.9(2.1-7.5)	0.169
> 45	43	5.3(1.9-8.1)		5.8(3.1-8.8)	
Tumor Size					
≤ 2 cm	50	3.7(1.5-6.8)	0.009*	3.2(1.5-5.2)	<0.001*
> 2 cm	48	6.3(3.5-8.1)		7.7(5.2-11.9)	
LNM					
Yes	62	5.1(3.5-7.7)	0.639	5.1(2.4-8.3)	0.565
No	36	5.3(1.6-7.2)		5.3(2.6-7.8)	
TNM Stage					
I+II	62	3.5(1.7-6.7)	<0.001*	4.0(2.0-6.4)	0.002*
III+IV	36	7.3(4.4-10.5)		7.3(3.5-11.3)	

*Indicated statistical significance (P<0.05).

^a^Median of relative expression according to HSCORE system, with 25th-75th percentile in parenthesis.

LNM, lymph node metastasis.

### Knockdown of PAK4 in PTC-derived cell lines retarded cellular proliferation, migration, and invasion

**Figure 2:**
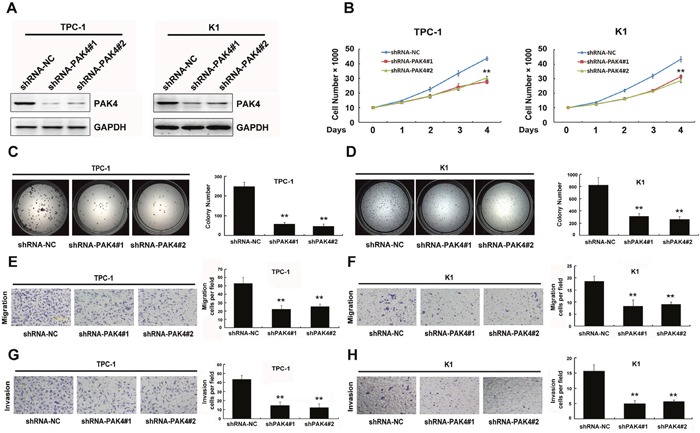
PAK4 knockdown in PTC-derived cell line retards cellular proliferation, migration and invasion **A**. Stable PAK4 knockdown in TPC-1 and K1 cell lines was detected by immunoblot analysis. TPC-1 and K1 cells were stably transduced with two different lentiviral vectors, shPAK4 or the non-targeting control shRNA (NC shRNA). **B** Proliferation was monitored by daily quantification of cell number for up to 4 days. ** *P* < 0.01. **C** and **D**. The colony forming assay showed that PAK4 knockdown inhibited cell growth in TPC-1 (C) and K1 (D) cells, ** *P* < 0.01. **E-H**. PAK4 knockdown retarded cellular migration and invasion in TPC-1 (E and G) and K1 **F** and **H**. cell lines, respectively. The results are presented as a mean ± SD of three independent experiments. **P < 0.01.

To characterize the function of PAK4 in PTC, basal PAK4 levels were reduced by RNAi-mediated knockdown in two PTC-derived cell lines, TPC-1 and K1. We found that two independent target sequences markedly decreased PAK4 levels than that observed with the control sequence in both cell lines (Figure 2A). Furthermore, PAK4 knockdown significantly inhibited cell growth (Figure 2B), colony formation (Figure 2C and 2D), cell migration (Figure 2E and 2F), and invasion (Figure 2G and 2H). In summary, these findings suggest that PAK4 expression is required for development and progression of PTC.

### TSH stimulated PAK4 activity through the TSHR/cAMP/PKA pathway

**Figure 3:**
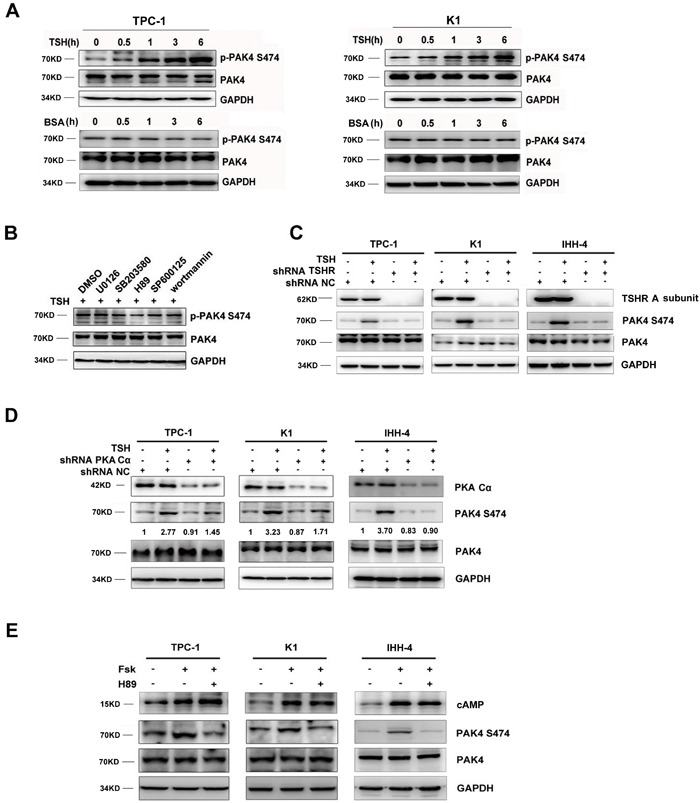
TSH stimulates PAK4 activity through the TSHR/cAMP/PKA pathway **A**. TSH stimulates PAK4 activity in a time-dependent manner. PAK4 and p-PAK4 levels were examined by western blot; BSA as a negative control. GAPDH was used an endogenous reference protein. **B**. PKA inhibitor (H89) significantly blocked PAK4 serine 474 phosphorylation by TSH though western blot. TSHR C. or PKA Cα D. knockdown inhibits TSH-induced PAK4 activation. Quantitative data (p-PAK4/total-PAK4 relative intensity) are shown. **E**. TPC-1, K1 and IHH-4 cells were treated with forskolin (20 μM) or forskolin plus H89 (10 μM), and p-PAK4 and PAK4 levels were examined by western blot.

In PTC-derived cell lines, TSH stimulated PAK4 phosphorylation at serine 474 at various time points after exposure to TSH than after exposure to BSA (Figure 3A). To identify the kinase responsible for this phosphorylation, we tested an array of kinase inhibitors: U0126 (MEK 1/2 inhibitor), SB203580 (p38 MAPK inhibitor), H89 (PKA inhibitor), SP600125 (c-Jun N terminal kinase inhibitor), and wortmannin (phosphoinositide 3-kinase inhibitor). Only the PKA inhibitor (H89) significantly blocked PAK4 serine 474 phosphorylation by TSH (Figure 3B). Furthermore, our results showed that TSH markedly increased levels of endogenous p-PAK4 in PTC-derived cells, while depletion of endogenous TSHR and PKA Cα by specific siRNAs reduced PAK4 phosphorylation following TSH exposure (Figures 3C and 3D). This suggests that TSH induced PAK4 phosphorylation in a TSHR and PKA-dependent manner. PTC-derived cells were treated with forskolin, a compound that stimulates adenylate cyclase activity and increases intracellular cAMP level. Forskolin treatment resulted in increased p-PAK4 levels, while H89 reduced the forskolin-induced p-PAK4 levels (Figure 3E). Taken together, these findings indicate that TSH stimulates PAK4 activity through the TSHR-cAMP-PKA pathway.

### Depletion of PAK4 and TSHR attenuated TSH-induced cellular proliferation in PTC

**Figure 4:**
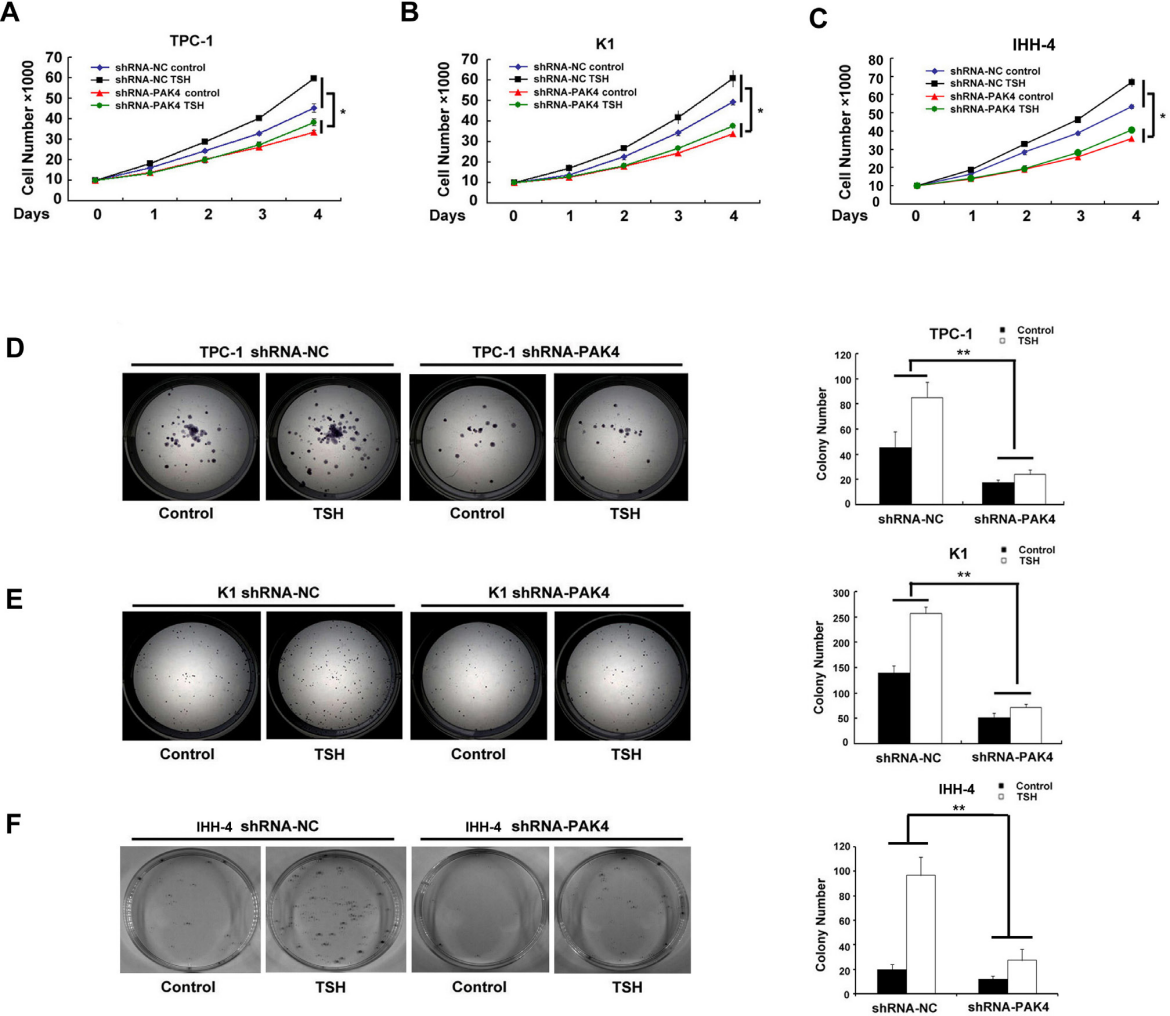
Depletion of PAK4 attenuates TSH-induced cellular proliferation TSH promotes TPC-1 **A**. K1 **B**. and IHH-4 **C**. cellular proliferation in a PAK4-dependent manner. Proliferation was monitored by counting cells daily for up to 4 days; * *P* < 0.05. Colony forming assay showed that TSH promoted cellular proliferation in TPC-1 D. K1 **E**. and IHH-4 **F**. cell lines in a PAK4-dependent manner, ** *P* < 0.01.

We demonstrated that TSH markedly enhances cell growth (Figures 4A-4C, Supplementary Figures 1A-1C) and colony formation in PTC-derived cell lines (Figures 4D-4F, Supplementary Figure 1G), whereas PAK4 and TSHR depletion significantly inhibited both the processes. Moreover, forskolin treatment increased cell growth (Supplementary Figures 1D-1F) and colony formation (Supplementary Figure 1H) in PTC-derived cell lines, whereas PAK4 depletion in these cell lines had the opposite effect. These results indicate that TSH promotes cellular growth and colony formation by the TSHR-cAMP-PKA-PAK4 pathway in PTC-derived cell lines.

### p-PAK4 levels correlate with TSH and PKA Cα levels in clinical PTC samples

**Figure 5:**
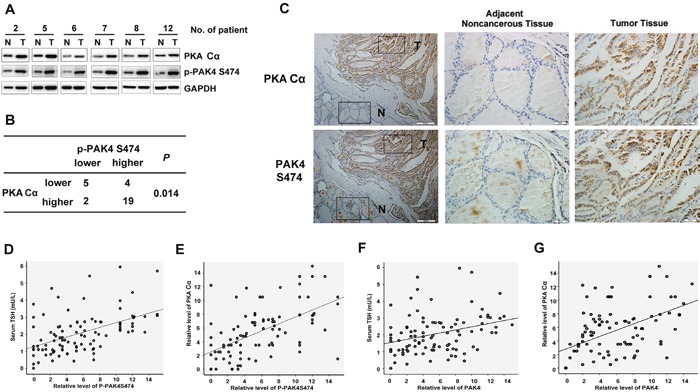
Correlation between levels of activated PAK4 and levels of TSH and PKA Cα in clinical samples of PTC **A**. Western blot analysis of PKA Cα and p-PAK4 in cancerous tissues from patients with PTC (T) and matched adjacent noncancerous tissues (N). **B**. Summary of the expression in tissues in (A) is shown, with tissues categorized by lower and higher expression. The expression of p-PAK4 and PKA Cα in Supplementary Figure 2 was analyzed with glyceraldehyde 3-phosphate dehydrogenase as the reference. In each N and T pair, the lower/higher expression in T, compared with N, is categorized as lower/higher expression. *P*-value was generated using the χ^2^-test. **C**. Immunohistochemical analyses of PKA Cα and p-PAK4 in tissues obtained from patients with PTC. The boxed areas in the left hand side images are magnified in the right hand side images. **D-G**. Immunohistochemical analyses of PKA Cα, p-PAK4 and PAK4 were scored in a semiquantitative manner according to both the intensity and the percentage of cells that were stained at each intensity in 98 PTC patients. P-PAK4 (D) and PAK4 (F) levels positively correlated with serum TSH levels in the sera of patients with PTC. Relative levels of p-PAK4 and PAK4 were plotted against TSH levels. P-PAK4 (E) and PAK4 (G) levels positively correlated with PKA Cα levels in PTC samples. Relative p-PAK4 and PAK4 levels were plotted against PKA Cα levels.

To further characterize the roles of TSH-PKA-PAK4 signaling in PTC and to confirm a functional link between PAK4 and PKA Cα, we explored the levels of PKA Cα and p-PAK4 in freshly frozen PTC tissues and matched adjacent noncancerous tissues from 30 patients with PTC (Supplementary Figure 2). The results revealed that PKA Cα levels correlated positively with the presence of p-PAK4 (*P* = 0.014) (Figures 5A and 5B).

Next, we used immunochemical staining of PKA Cα and p-PAK4 in a large number of clinical section samples. We found that PKA Cα and p-PAK4 levels were significantly upregulated in PTC-derived tissues than in adjacent noncancerous tissues (Figure 5C, Table 1). Furthermore, we determined p-PAK4 and PAK4 levels in PTC samples and plotted them against levels of TSH and PKA Cα. As shown in Figures 5D-5G, there was a positive correlation between these two variables (Figure 5D, *r* = 0.502, *P* < 0.001; Figure 5E, *r* = 0.596, *P* < 0.001; Figure 5F, *r* = 0.346, *P* < 0.001; Figure 5G, *r* = 0.504, *P* < 0.001, Spearman's rank correlation test). These results were consistent with our findings that TSH upregulated p-PAK4 through the PKA pathway in PTC-derived cell lines.

## DISCUSSION

We report that PAK4 and p-PAK4 were upregulated in clinical PTC samples. More importantly, increased levels and activity of PAK4 were found to significantly correlate with tumor size and tumor TNM stage. These findings suggest that PAK4 is associated with an aggressive form of clinical PTC—a novel finding.

PAKs 1-4 and PAK6 are overexpressed in human thyroid cancer cell lines as well as in samples from patients with thyroid cancer. Among the group I PAKs, siRNA-mediated knockdown of only PAK1 reduced migration significantly in thyroid cancer cell lines, but an additional role for PAK4 was not excluded [25]. In the present study, we demonstrate that levels and activity of PAK4 are high in PTC samples. Furthermore, PAK4 knockdown inhibited cellular proliferation, migration, and invasion in TPC-1 and K1 PTC cell lines.

PAK4 activity can be induced in a Rho GTPase-independent manner. PAK4 is upregulated by progesterone and TNF-α in endometrial cell lines [32] and is activated by HGF via phosphoinositide 3 kinase (PI3K)[4]. Moreover, expression of PAK4 is also induced by follicle stimulating hormone (FSH) [22] and by human chorionic gonadotropin (hCG) [33]. In this study, we demonstrate that TSH increases endogenous p-PAK4 levels, but not PAK4 expression in PTC cells. Since FSH and hCG can both induce TSH expression, why does TSH upregulate PAK4 phosphorylation rather than expression? The mechanism of this phenomenon is still unclear. Maybe it is because that FSH and hCG induced PAK4 expression is not TSH dependent.

In conclusion, the current study demonstrates that increased levels and activity of PAK4 in PTC can be associated with disease progression. Furthermore, TSH-induced increase in PAK4 activity was found to promote the invasive potential of thyroid cancer cells, thereby identifying a novel role of TSH signaling in prognosis of PTC. Further study of the TSH-PAK4 signaling system may provide promising new therapeutic targets for PTC.

## MATERIALS AND METHODS

### Cell lines, production of lentiviral particles and establishment of stable cell lines

Human PTC cell lines, TPC-1, K1 and IHH-4 were used; TPC-1 was a gift from Bryan R. Haugen (Division of Endocrinology, Diabetes and Metabolism, University of Colorado Denver, Aurora, CO); K1 was purchased from The Health Protection Agency Culture Collections, UK. The human thyroid cancer cell line IHH-4 was established from a 75-year-old man with a papillary thyroid carcinoma and was obtained from Health Science Research Resources Bank (Osaka, JAPAN). IHH-4 cells were maintained in a 1:1 mixture of DMEM and RPMI-1640 supplemented with 10% fetal bovine serum (Invitrogen). TPC-1 and K1 cells were cultured in RPMI 1640 medium with 10% fetal bovine serum in a humidified incubator at 37 °C and 5% CO_2_. Cells were starved in basal medium (devoid of growth factors and serum) for 48 h before stimulation with TSH (10 nM, approximately equal to 2 mU/mL). PAK4- and PAK4-RNAi-lentiviral vectors were purchased from Shanghai GeneChem Company (Shanghai, China). The PAK4 #1 sequence was 5′-GGATGAACGAGGAGCAGAT-3′; the PAK4 #2 sequence was 5′-CTTCATCAAGATTGGCGAG-3′ and the shRNA control sequence was 5′-TTCTCCGAACGTGTCACGTtt-3′. TPC-1 and K1 cells were cultured in a 12-well plate and transfected with lentivirus with 3 mg/ml polybrene. The PKA Cα and TSHR gene–specific siRNA sequence were 5′-AAGTGGTTTGCCACAACTGAC-3′and5′-TCCAAAGAACAGCACTGAT-3′.

### Western blot analysis

Cells were starved and pretreated with U0126 (5 μM), SB203580 (5 μM), H89 (10 μM), SP600125 (5 μM) or wortmannin (0.5 μM) for 1 h and then stimulated with 10 nM TSH for 60 min. Cells were lysed in lysis buffer (20 mM Tris-HCl, 150 mM NaCl, 2 mM EDTA and 1% Triton-X 100) with a protease inhibitor cocktail for 30 min at 4°C. Lysates were quantified using a BCA protein assay kit (Pierce) according to the manufacturer's instructions. Proteins in the lysate were separated by 10% SDS-PAGE and transferred to a PVDF membrane (Millipore). After blocking with 5% skimmed milk, the membranes were incubated with anti-PAK4 (1:1000), anti-p-PAK4 (1:1000), anti-PKA Cα(1:1000) (Cell Signaling Technology, Beverly, MA, USA), anti-TSHR (1:1000) (Santa Cruz), cAMP (1:1000) (Sigma) and anti-GAPDH (1:2000) (GenScript Corporation, Nanjing, China) antibodies. U0126, SB203580, H89, SP600125, wortmannin, and forskolin were purchased from BioTime Technology.

### Colony formation assay

Cellular proliferation potential was assessed by colony formation. Briefly, 400 cells were plated in six-well plates and incubated at 37 °C in a 5% CO_2_ incubator. After 2 weeks of culture, cells were stained with crystal violet and colonies were counted. The data is represented as a mean ± SD from 3 independent experiments.

### Cell migration and invasion assays

Transwell cell migration and Matrigel invasion assays were performed using Boyden chambers with polycarbonate nucleopore membranes. Precoated filters (6.5 mm in diameter, 8 mm pore size; Matrigel 100 mg/cm^2^) were rehydrated with 100 μl medium. In the upper part of each chamber, 1 × 10^5^ cells were seeded in 100 μl serum-free Dulbecco's modified Eagle's medium supplemented with 0.1% bovine serum albumin, while the lower compartments were filled with 600 μl Dulbecco's modified Eagle's medium containing 10% serum as described previously [6].

### Patients and tissue specimens

Specimens from the thyroid were obtained from 98 Chinese patients who had PTC, which included 55 patients of reproductive age (18 – 45 years old) and 43 of advanced reproductive age (> 45 years old). All patients had a clinical duration of less than 3 years and had been admitted to the hospital for standard thyroidectomies from 2011 to 2013; those who underwent secondary surgery for PTC were excluded. Diagnoses were confirmed through histopathological examination. None of the patients had a history of familial thyroid cancer or neck external irradiation. TNM stage was assessed according to the tumor, node, and metastasis system classification proposed by the American joint committee on cancer (7th edition).

### Immunohistochemistry

Paraffin-embedded PTC tissues were obtained from the First Hospital of China Medical University. Five-micrometer-thick consecutive sections were excised and mounted on glass slides. The slides were deparaffinized and rehydrated prior to antigen retrieval and endogenous peroxidase blocking. The sections were then washed three times in 0.01 mol/L PBS for 5 minutes each and blocked for 1 h in 5% normal goat serum. The sections were exposed to anti-PAK4 (1:100), anti-p-PAK4 (1:100) and anti-PKA Cα (1:100) antibodies overnight at 4°C. After brief washes in 0.01 mol/L PBS, sections were incubated in 0.01 mol/L PBS containing horseradish peroxidase-conjugated goat anti-rabbit immunoglobulin G (1:200) for 2 h, followed by development with 0.003% H_2_O_2_ and 0.03% 3, 3′-diaminobenzidine in 0.05 mol/L Tris-HCl.

The immunostained sections were reviewed by two authors who had no knowledge of the patients’ clinical status. Five areas that were selected at random were scored. All sections were scored in a semiquantitative manner according to a previously described method, which reflects both the intensity and the percentage of cells that were stained at each intensity [34]. Intensity was classified as 0 (no staining), + 1 (weak staining), + 2 (distinct staining) or + 3 (very strong staining). A value designated as the ‘HSCORE’ was obtained for each slide by using the following algorithm: HSCORE = ∑ (I × PC), where I and PC represent the staining intensity and the percentage of cells that stain at each intensity, respectively.

### Statistical analysis

All statistical analyses, including Student's *t*-test and Mann-Whitney U test (non-parametric), were performed using the SPSS 16.0 software. Results were considered significant if *P* < 0.05 and the data are presented as a mean ± SD from at least three independent experiments. Association between variables was analyzed using Spearman's rank correlation test.

## SUPPLEMENTARY MATERIALS FIGURES


