# Supplementary material for: P21-activated kinase 4 involves TSH induced papillary thyroid cancer cell proliferation
Source: Oncotarget. 2017 Feb 4;8(15):24882–91. doi: 10.18632/oncotarget.15079 (PMC5421896; doi:10.18632/oncotarget.15079)
Supplement: Supplementary file 1 [file oncotarget-08-24882-s001.pdf]

# P21-activated kinase 4 involves TSH induced papillary thyroid cancer cell proliferation

## SUPPLEMENTARY FIGURES

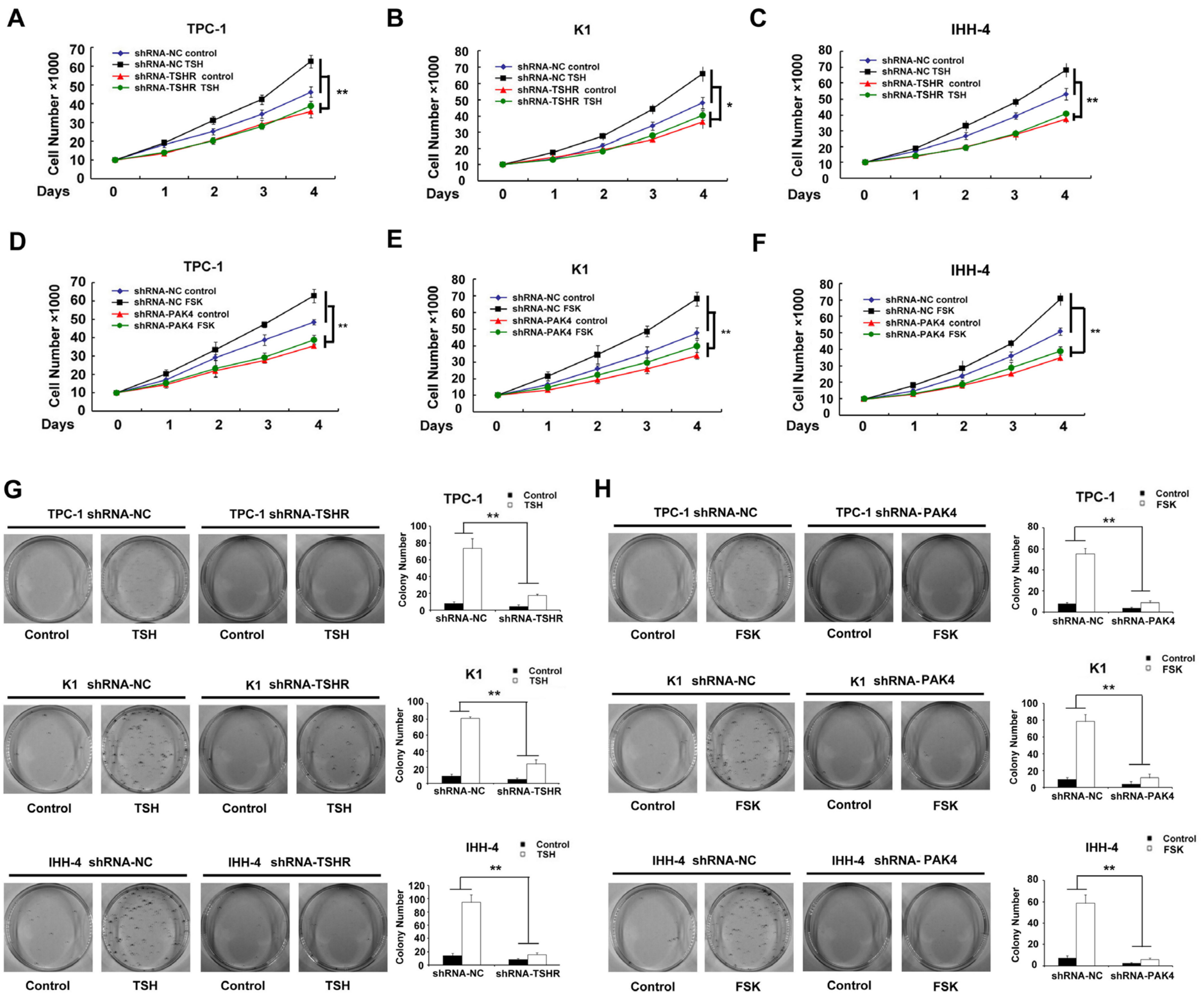


**Supplementary Figure 1: A-C.** TSH promotes TPC-1 (A), K1 (B) and IHH-4 (C) cellular proliferation in a TSHR-dependent manner. Proliferation was monitored by counting cells daily for up to 4 days; * $P < 0.05$, ** $P < 0.01$. **D-F.** FSK promotes TPC-1 (D), K1 (E) and IHH-4 (F) cellular proliferation in a PAK4-dependent manner. Proliferation was monitored by counting cells daily for up to 4 days; ** $P < 0.01$. **G.** Colony forming assay showed that TSH promoted cellular proliferation in TPC-1, K1 and IHH-4 cell lines in a TSHR-dependent manner, ** $P < 0.01$. **H.** Colony forming assay showed that FSK promoted cellular proliferation in TPC-1, K1 and IHH-4 cell lines in a PAK4-dependent manner, ** $P < 0.01$.

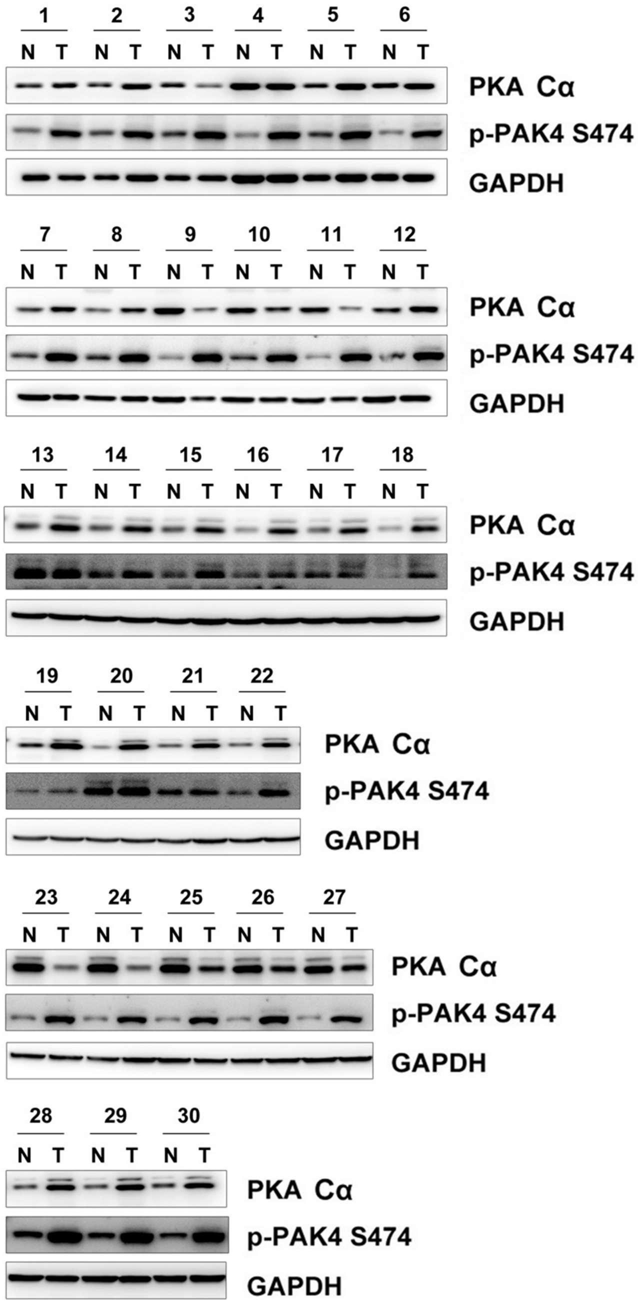


**Supplementary Figure 2: Western blot analysis of PKA Cα and p-PAK4 in cancerous tissues from patients with PTC (T) and matched adjacent noncancerous tissues (N).**
